# Abnormal Magnetic Resonance Imaging Patterns in Patients with Neuropsychiatric Disorders Due to Anti-NMDA Receptor Encephalitis: A Comparative Study

**DOI:** 10.3390/diagnostics16030391

**Published:** 2026-01-26

**Authors:** Miguel Restrepo-Martinez, Roger Carrillo-Mezo, Abel Medina-Islas, Manuel Ricardo Barojas-Alvarez, Marcela Otero-Cisneros, Francisco M. Martínez-Carrillo, Mariana Espínola-Nadurille, Verónica Rivas-Alonso, Victoria Martínez-Ángeles, Arely Juárez-Jaramillo, José de Jesús Flores-Rivera, Elizabeth Varela-Blanco, Jesús Ramirez-Bermudez

**Affiliations:** 1 Neuropsychiatry Unit, National Institute of Neurology and Neurosurgery, Mexico City 14269, Mexico; mrm87356@hotmail.com (M.R.-M.); manuelricardobarojasalvarez@gmail.com (M.R.B.-A.); cisneros_marcela@hotmail.com (M.O.-C.); francisco.martinez@innn.edu.mx (F.M.M.-C.); mariana.espinola@innn.edu.mx (M.E.-N.); dra.mtz.ang.victoria@gmail.com (V.M.-Á.); cynthia.arely.6@gmail.com (A.J.-J.); 2 Neuropsychiatry Unit, Americas Clinic, Medellín 050001, Colombia; 3 Neuroradiology Service, National Institute of Neurology and Neurosurgery, Mexico City 14269, Mexico; mayroger@hotmail.com (R.C.-M.); abel_mire@hotmail.com (A.M.-I.); elizabeth.varela@innn.edu.mx (E.V.-B.); 4 Neurology Department, National Institute of Neurology and Neurosurgery, Mexico City 14269, Mexico; veronica.rivas@innn.edu.mx (V.R.-A.); jflores.rivera@gmail.com (J.d.J.F.-R.); 5 School of Medicine, National Autonomous University of Mexico, Mexico City 04510, Mexico

**Keywords:** anti-NMDA receptor encephalitis, T2-FLAIR, MRI, meningeal enhancement, pachymeningeal enhancement, leptomeningeal enhancement, psychosis, catatonia, delirium

## Abstract

**Background:** Brain MRI abnormalities in anti-NMDA receptor encephalitis (ANMDARE) are classically described in limbic structures, particularly the medial temporal lobe. Paralimbic, neocortical, and meningeal abnormalities have been less consistently reported. **Objective:** The objective was to evaluate the diagnostic value of brain MRI abnormalities in patients with definite ANMDARE. **Methods:** We conducted a case–control study including 115 patients with ANMDARE and 115 controls with primary psychotic disorders or antibody-negative autoimmune encephalitis. Structural MRI studies were systematically reviewed by an expert neuroradiologist blinded to clinical diagnosis. **Results:** ANMDARE patients were younger and more frequently presented with seizures, dyskinesia, severe neuropsychiatric disturbances, abnormal cerebrospinal fluid and EEG findings, and worse outcomes, including mortality. T2-T2-FLAIR abnormalities commonly involved medial temporal limbic structures, paralimbic regions (anterior cingulate and insular cortices), and neocortical areas (parieto-occipital cortices). Pachymeningeal enhancement was observed in 26.1% of patients. MRI findings clearly differentiated ANMDARE from primary psychotic disorders but largely overlapped with antibody-negative autoimmune encephalitis, except for limited parietal and occipital differences. **Conclusions:** T2-FLAIR MRI abnormalities involving medial temporal, paralimbic, and posterior neocortical regions are common in ANMDARE. Pachymeningeal enhancement is not rare. While useful for distinguishing ANMDARE from primary psychotic disorders, a substantial overlap with antibody-negative autoimmune encephalitis was observed.

## 1. Introduction

Anti-N-methyl-D-aspartate receptor encephalitis (ANMDARE) is a frequent and severe autoimmune-mediated disorder [1], characterized by prominent neuropsychiatric manifestations [2]. Although it is ultimately diagnosed by the presence of IgG autoantibodies against the NR1 subunit of the NMDA receptor (NMDAR) in CSF, its clinical suspicion is based on psychiatric and neurological manifestations and compatible abnormalities on brain MRI, EEG, and CSF analysis [2].

While brain MRI is a crucial exam in the work-up of autoimmune encephalitis [2], abnormalities on brain MRI are not part of the ANMDARE diagnostic criteria, given the low frequency of specific MRI patterns in this population [3,4]. Whether high or low, the prevalence of MRI abnormalities varies widely across studies. A recent systematic review, which included 55 studies reporting findings on the frequency of abnormal MRI findings in ANMDARE, found that in the acute phase, 440 (37.7%) out of 1167 patients showed abnormal MRIs (CI 35.0–40.5, 95%) [5]. Notably, of these studies, the highest frequency of abnormal brain MRI observed was 83.3%, and the lowest was 11.1% [6], suggesting significant inconsistencies and heterogeneity not only in the definition of the reported MRI abnormalities, but also possibly in the timing, sequences used, and cohort selection [5].

So far, brain MRI abnormalities in anti-NMDA receptor encephalitis (ANMDARE) have been described primarily in limbic structures, particularly the medial temporal lobe [2,7]. Paralimbic, neocortical, and meningeal abnormalities have been less consistently reported. In recent years, various patterns of brain MRI abnormalities or lesions have been described, supporting the notion that the distribution is more widespread than previously assumed [4,8,9]. A recent retrospective cohort study, involving 255 patients with ANMDARE, found that 37 (14.5%) had limbic hyperintensities, and 41 (16.1%) had extralimbic lesions; 10 patients had overlapping demyelinating syndromes as multiple sclerosis (MS), neuromyelitis optica spectrum disorder (NMOSD), or myelin oligodendrocyte glycoprotein–associated disorder (MOGAD) [10]. Significantly, some abnormal brain MRI findings in ANMDARE have been associated with poor clinical outcomes and are criteria for the One-Year Functional Status (NEOS) anti-NMDAR encephalitis score [9,11].

Given the described clinical, diagnostic, and prognostic implications of brain MRI abnormalities in ANMDARE and the inconsistencies and knowledge gap in the field, we have conducted a dedicated MRI study in patients with ANMDARE admitted to the National Institute of Neurology and Neurosurgery (NINN) of Mexico City, systematically investigating brain MRI features and comparing them with controls with similar neuropsychiatric manifestations. Thus, the authors aimed to characterize brain MRI abnormalities in patients with ANMDARE and their associated clinical implications.

## 2. Materials and Methods

Design. A nested case–control study was conducted in a cohort of patients with suspected autoimmune encephalitis treated at the National Institute of Neurology and Neurosurgery of Mexico (NINN). This study was conducted in accordance with the World Medical Association Declaration of Helsinki and was approved by the NINN institutional review board and ethics committee under Protocol No. 118/19.

Patients. The study population included patients admitted to the NINN from 2013 to 2018 who were assessed for suspected autoimmune encephalitis. These patients fulfilled the criteria for possible autoimmune encephalitis by Graus et al., for possible autoimmune psychosis by Pollak et al., or had a first psychotic episode in the presence of at least one red flag to suspect this entity [2,7,12]. Among red flags, we included a history of flu-like prodrome, rapid onset of psychotic symptoms, severe and disproportionate cognitive dysfunction, delirium, catatonia, seizures, dyskinetic movements, and severe autonomic dysfunction [2,7,12]. Sampling was consecutive according to the inclusion and exclusion criteria. Patients with a positive determination of NMDA receptor antibodies in CSF, who fulfilled the Graus’ criteria for definite anti-NMDA receptor encephalitis, were selected as cases. Patients with neuropsychiatric disturbances and suspected autoimmune encephalitis who were finally classified as not having ANMDARE after diagnostic studies were selected as controls.

Sociodemographic and clinical variables. We collected sociodemographic data and relevant clinical variables, including psychiatric and cognitive symptoms, speech disturbances, motor signs, seizures, altered level of consciousness, autonomic imbalance, hypoventilation, and others that emerged in our subjects during their admission, hospital stay, and at discharge. A detailed description of the clinical measures has been provided elsewhere [13].

Diagnostic studies. On admission, CSF cytochemical analysis and EEG were obtained in all patients as part of the diagnostic routine. If the patients fulfilled Graus’ criteria for possible autoimmune encephalitis, or Pollak’s probable autoimmune psychosis, a CSF sample was taken from all patients to look for antibodies against the NR1 subunit of N-methyl-D-aspartate glutamate receptor, which were processed at Labco Nous Diagnostics, Barcelona, Spain, with rat brain immunohistochemistry and cell-based assays with NMDA expressing cells. Several tests were performed on admission and were negative in all patients, as follows: Tests for bacteria (including cultures for *M. tuberculosis* and *Cryptococcus neoformans*) and HIV; CSF adenosine deaminase; tests for systemic autoimmune diseases (anti-double-stranded DNA, antinuclear antibodies, antineutrophil cytoplasmic antibodies, anti-beta 2 glycoprotein antibodies, and antiphospholipid antibodies) and thyroid disease. Viral CSF PCR results for Herpes simplex types 1 and 2, Cytomegalovirus, Epstein–Barr, Varicella zoster, Human herpes types 6, 7, and 8, Enterovirus, Toxoplasma, Parvovirus B19, and Lymphocytic choriomeningitis virus were also negative in the current episode of all patients. Reasonably, these tests excluded other disorders.

MRI studies. An experienced technician collected the MRI data within 1–2 weeks after hospital admission in all patients. All MRIs were performed on a MAGNETOM Skyra-Siemens 3T MRI (SIEMENS Healthcare Erlangen, Germany). Axial T2-weighted image (T2WI), T1-weighted image (T1WI), and the fluid-attenuated inversion recovery image (T2-FLAIR) were included. Contrast-enhanced studies were obtained in all patients using intravenous Gadoteridol (ProHance, Bracco Diagnostics, manufactured by BIPSO GmbH, Singen, Baden-Wurtemberg, Germany). An expert neuroradiologist conducted a systematic evaluation of all brain MRIs using a standardized data collection format and was blinded to patients’ clinical classification. The collection format for brain MRI included the assessment of the medial temporal region, including the amygdala, the hippocampus, and the parahippocampal gyrus, as well as the following regions: the thalamus, the striatum, the insular and cingulate cortices (anterior, mid, and posterior), and prefrontal, parietal, lateral temporal, and occipital cortices. In each case, the presence of pachymeningeal and leptomeningeal enhancement was assessed. Finally, the medial temporal atrophy scale and the generalized cortical atrophy scale were scored in all patients.

Statistical analysis. Data analysis was performed with the SPSS software, version 21. For nominal variables, absolute values and proportions were obtained. Quantitative variables were described using measures of central tendency and their respective measures of dispersion. Normality tests (Kolmogorov–Smirnov test) were obtained for quantitative variables. To assess the diagnostic value of the clinical and MRI variables, we estimated positive likelihood ratios (LR+) with 95% confidence intervals, treating anti-NMDAR encephalitis as the target condition and the control group as the reference. We used the standard definition: positive LR = Sensitivity/1 − Specificity. For further analysis, we used inferential statistics, including Pearson’s chi-square test or Fisher’s test for categorical variables, and the *t*-test or Mann–Whitney U test for continuous variables. Because multiple comparisons were made, we used the Bonferroni correction to control type I error.

## 3. Results

### 3.1. Characteristics of the Sample

254 patients were admitted to the National Institute of Neurology and Neurosurgery with a suspicion of autoimmune encephalitis. Of these, 115 had positive NMDA receptor antibodies in CSF and fulfilled Graus’ diagnostic criteria for definite anti-NMDA receptor encephalitis. These patients were selected as cases. 139 patients were not diagnosed as having anti-NMDA receptor encephalitis. Of these, 24 patients were excluded from this study as they were diagnosed as having specific neurological diseases with particular brain imaging abnormalities (viral encephalitis, *n* = 5; prion disease, *n* = 4; anti-LGI1 encephalitis, *n* = 3; systemic lupus erythematosus, *n* = 3; epilepsy, *n* = 3; bacterial infection, *n* = 2; anti-AMPA encephalitis, *n* = 1; COVID encephalopathy, *n* = 1; metabolic encephalopathy, *n* = 1). This exclusion criterion was used to reduce the heterogeneity in the sample. Finally, the control group consisted of 115 patients without anti-NMDA receptor encephalitis (after the diagnostic assessment, 75 were diagnosed as having a primary psychotic disorder, and 40 patients had a final diagnosis of probable autoimmune encephalitis with a negative determination of NMDAR antibodies).

### 3.2. A Comparative Analysis of Sociodemographic and Clinical Variables

Patients with definite ANMDARE were younger (34.73 ± 15.27 years old vs. 28.30 ± 10.63, *p* < 0.001, *t*-test). There was no significant difference between groups regarding the proportion of female sex (47.8 vs. 49.6%, *p* = 0.792, Pearson’s chi-square test). As may be seen in Table 1, patients with definite ANMDARE presented more seizures and dyskinesia than patients without anti-NMDAR encephalitis. We also observed significant differences in the neuropsychiatric profile. Patients with definite ANMDARE had more features of psychomotor agitation, severe cognitive dysfunction, and catatonia. Also, the outcome of the patients was worse in patients with ANMDARE: this group had a higher rate of coma state, ICU requirement, and mortality. Finally, abnormalities of CSF, EEG and MRI were more common in the definite ANMDARE group. The classification of each patient as having an abnormal MRI or not was based on the clinical judgment of the neuroradiologist. The sensitivity of an abnormal MRI was 56%, and the specificity was 73%. As expected, EEG was the most sensitive measure (87.6%), whereas CSF was the most specific (84.3%).

### 3.3. The Assessment of Brain MRI Findings

**Figure 1:**
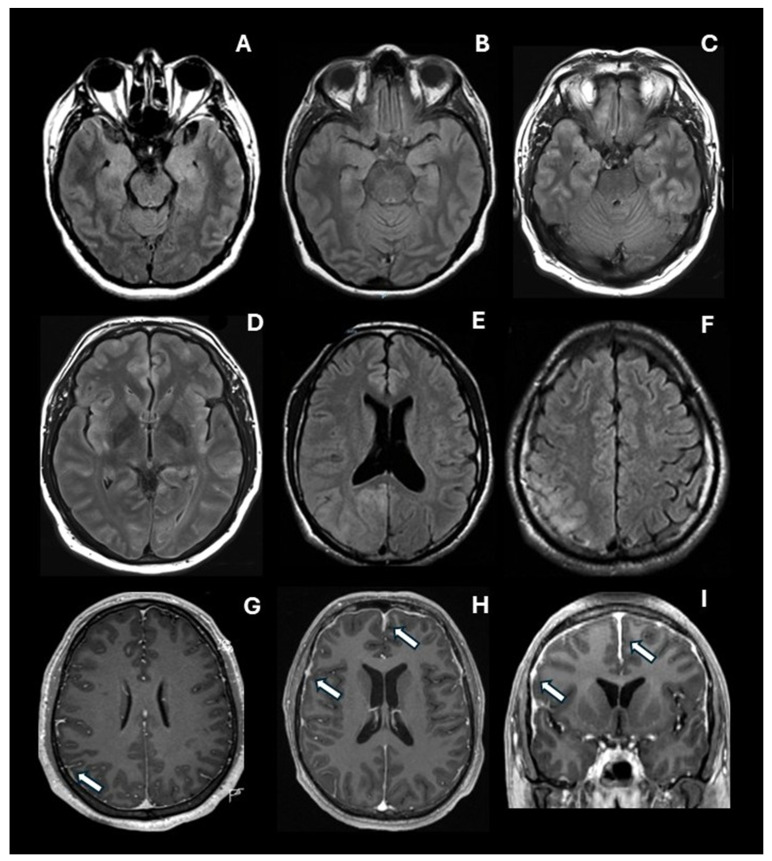
Brain MRI abnormalities in anti-NMDA receptor encephalitis. Limbic, paralimbic, and neocortical abnormalities found in the T2 T2-FLAIR sequence are depicted. Bilateral hyperintense signal can be seen: (**A**) in the uncus and the amygdala, bilaterally; (**B**) in the hippocampus, bilaterally; (**C**) in the medial aspects of the temporal lobe, bilaterally, and in the left lateral temporal cortex; (**D**) in the insular cortex, and the anterior cingulate cortex, bilaterally; (**E**) in the right anterior cingulate cortex and retrosplenial cortex; and (**F**) in the right lateral parietal cortex. Also, representative images of contrasted T1 sequence with leptomeningeal (**G**) and pachymeningeal (**H**,**I**) enhancement are shown and highlighted through the arrows.

**Figure 2:**
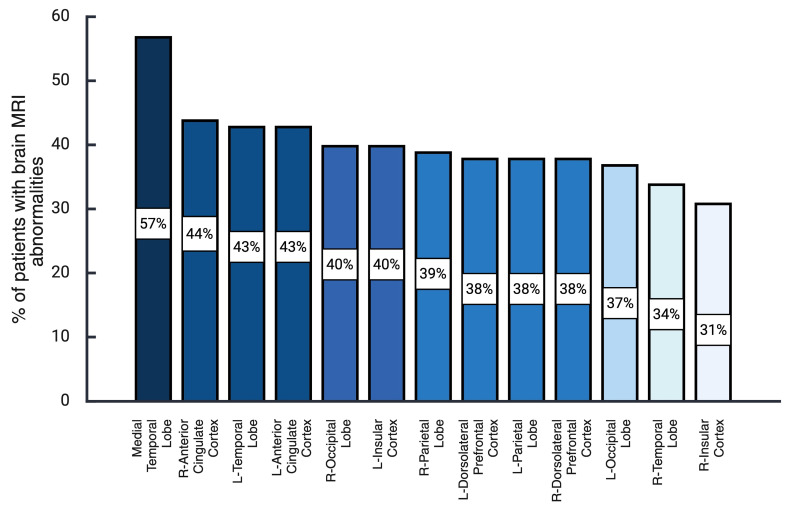
Frequency and distribution of brain MRI abnormalities in anti-NMDAR encephalitis. Created in https://BioRender.com, accesed on 15 January 2026.

Through the expert neuroradiologist’s assessment, blinded to the patient’s diagnosis, a profile of brain abnormalities was observed in the T2-FLAIR sequence. Some characteristic abnormalities are depicted in Figure 1, including hyperintense images in limbic, paralimbic, and neocortical structures. Also, representative images of pachymeningeal and leptomeningeal enhancement are presented. As may be seen in Figure 2, the most frequent abnormalities were observed in the medial temporal lobe (56.5%), the right (44.3%) and the left (42.6%) anterior cingulate cortex, the left lateral temporal cortex (42.6%), the left insular cortex (40.0%), and the right occipital cortex (40.0%).

As may be seen in Table 2, the most substantial differences between cases and controls, which were statistically significant after Bonferroni correction for multiple comparisons (0.05/34 = 0.0014), were observed in the right occipital cortex (LR+ 4.18), the left occipital cortex (LR+ 3.50), the left parietal cortex (LR+ 2.75), the right anterior cingulate cortex (LR+ 2.68), the left insular cortex (LR+ 2.56), the right parietal cortex (LR+ 2.50), and the left anterior cingulate cortex (LR+ 2.45), and the medial temporal lobe (LR+ 2.13).

### 3.4. Subanalysis of Patients with Anti-NMDA Receptor Encephalitis vs. Patients with a Negative Determination of NMDA Receptor Antibodies

As our control group comprises patients with two types of pathology, we analyzed each group separately. We focused only on the variables that were significantly related to ANMDARE according to the previous analysis (presented in Table 2). As may be seen in Table 3, the frequency of abnormalities is similar in patients with ANMDARE as compared to patients with a negative determination of NMDA receptor antibodies, except for the parietal cortex in the left hemisphere (*p* = 0.035, Pearson’s chi-square test), and the occipital cortex in the right hemisphere (*p* = 0.010, Pearson’s chi-square test). Also, we found no significant differences regarding the frequency of pachymeningeal enhancement.

### 3.5. Subanalysis of Patients with Anti-NMDA Receptor Encephalitis vs. Patients with a Primary Psychotic Disorder

As may be seen in Table 3, the frequency of abnormalities in patients with ANMDARE was significantly higher than the frequency observed in patients with a primary psychotic disorder in all of the selected brain regions (*p* < 0.001, Pearson’s chi-square test). Also, there was a significant difference in the frequency of pachymeningeal enhancement (26.1% vs. 1.3%, *p* = 0.01, Pearson’s chi-square test).

## 4. Discussion

In the present study, we included 115 patients with a definite ANMDARE and compared them with 115 patients in a control group. Patients in the ANMDARE group had a high frequency of psychotic symptoms, psychomotor agitation, and catatonia. However, the most frequent neuropsychiatric abnormality was severe, disproportionate cognitive dysfunction. A more detailed account of cognitive abnormalities in our sample of anti-NMDA receptor encephalitis showed that global cognitive performance improves significantly one year after hospital discharge, although mild to moderate executive dysfunction may still be observed [14]. Regarding the brain imaging studies, patients with ANMDARE showed a significantly higher rate of MRI abnormalities across multiple limbic, paralimbic, and neocortical regions on the T2-FLAIR sequence. Similarly, the frequency of meningeal enhancement was higher in patients with ANMDARE, a finding that has been rarely reported in previous studies. Some examples of the characteristic brain MRI abnormalities observed in our patients with ANMDARE are shown in Figure 1.

### 4.1. Abnormal Brain MRI in ANMDARE

Previous studies in ANMDARE suggest that abnormal brain MRI findings in clinical practice are present in fewer than half of patients [3,6], with some studies reporting frequencies as low as 11% and others as high as approximately 80% [5]. In the largest cohort of patients with ANMDARE reported to date, Titulaer et al. found that only 33% of patients had abnormal brain MRIs at disease onset [3]. However, in a smaller cohort of 44 patients with ANMDARE, Irani et al. found that brain imaging was normal on initial MRIs in 39/44 (89%) and remained normal on subsequent examinations in 34/44 (77%) [6]. More recently, Wang et al., in a Chinese cohort of 106 patients with ANMDARE, found that 54 (50.9%) had abnormal or atypical brain MRI findings. Still, only 20 (37%) showed hyperintense signals on T2-FLAIR sequences, mainly in limbic and paralimbic cortices [8]. The other 34 patients showed “atypical” findings, such as meningeal enhancement, enlarged temporal horns of the lateral ventricles, pituitary lesions, and non-specific cortical and periventricular white matter lesions [8].

In the present study, an abnormal MRI was found in 56.5% of patients with ANMDARE, which is higher than most of the abovementioned studies [5], probably due to increased sensitivity resulting from a broader definition of “abnormal MRI” that extends beyond the classic mesial temporal involvement included in the diagnostic criteria for possible autoimmune encephalitis, as recommended by Graus et al. [2]. These results need to be interpreted in context. As shown in Table 1, the highest specificity (84.3%) in our study was achieved by an abnormal CSF result, although this measure showed the lowest sensitivity (56.5%). MRI studies had a similar likelihood ratio (2.10) to EEG studies. However, EEG demonstrated higher sensitivity (87.6%), whereas MRI showed higher specificity (73%). A more detailed analysis of abnormal EEG patterns in our sample of anti-NMDA receptor encephalitis revealed that diffuse slowing was the most frequent abnormality (sensitivity 75.7%), while the extreme delta brush pattern was the most specific feature (specificity 91.2%). After adjustment for confounders using logistic regression analysis, an abnormal EEG was strongly associated with anti-NMDA receptor encephalitis [15].

### 4.2. What Are the Most Frequently Involved Brain Structures in ANMDARE?

Most previous analyses of brain MRI of patients with ANMDARE have found that the most compromised brain areas are those related to limbic and paralimbic cortices. Extralimbic involvement, however, has also been reported [5]. In the study by Wang et al., the authors found T2 or T2-FLAIR signal hyperintensities mainly in the hippocampi, cerebellar and cerebral cortices, the insular cortex, and the basal ganglia [8]. Similarly, Zhang et al., in a study including 53 patients with ANMDARE, described 4 different types of MRI involvement at the onset phase (type 1: normal MR imaging findings; type 2: only hippocampal lesions; type 3: lesions not involving the hippocampus; and type 4: lesions in both the hippocampus and other brain areas). Around, 47% presented with abnormal MRI results: 28% (7/25) showed lesions in the hippocampus only; 28% (7/25) showed lesions in areas not involving the hippocampus, such as the frontal lobe, cingulate gyrus, corpus callosum, insula, thalamus, and brain stem; and 44% (11/25) showed lesions in both the hippocampus and other brain areas. In total, hippocampal lesions were the most common MRI abnormality detected in this sample [9]. In Bacci et al.’s systematic review, the most commonly reported MRI abnormalities in ANMDARE were T2/T2-FLAIR hyperintensity in the temporal lobes, reported in 91 cases; 53 of 91 were reported as medial temporal involvement [5]. However, other frequently reported sites of hyperintensities were the frontal lobes, hippocampus, periventricular region, and cerebellum [5].

**Figure 3:**
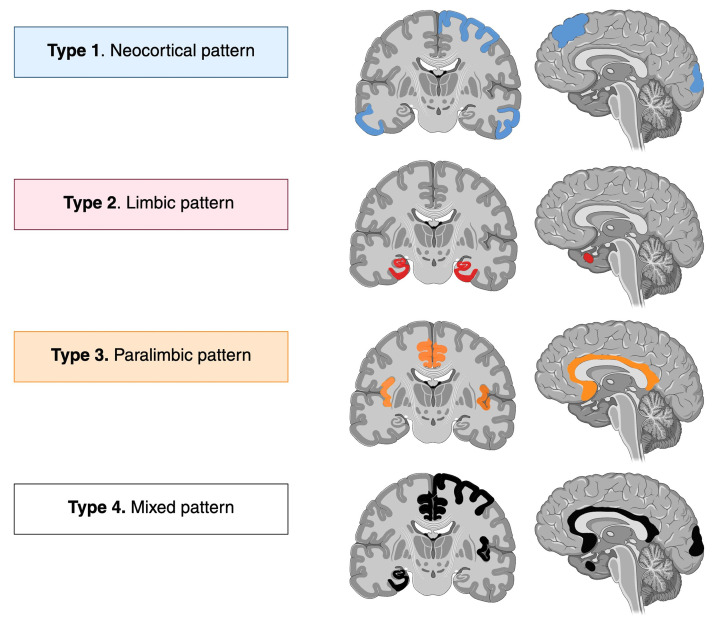
Topographic patterns of brain MRI abnormalities in ANMDARE. Schematic representation of the most frequent MRI patterns identified in the ANMDARE cohort. Type 1 was a neocortical pattern characterized by preferential involvement of cortical structures in the frontal, temporal, parietal and occipital lobes. Type 2 was a limbic pattern characterized by preferential involvement of mesial temporal and limbic structures, including the hippocampus and amygdala. Type 3 was a paralimbic pattern with involvement of mesocortical cortices, including the cingulate and insular cortices. Type 4 was a mixed pattern, as brain MRI patterns are not mutually exclusive. Created in https://BioRender.com, accesed on 15 January 2026.

In the present study, patients with ANMDARE showed a significantly higher frequency of MRI abnormalities across multiple limbic, paralimbic, and neocortical regions compared to controls. These patterns have been summarized in Figure 3. Most abnormalities were bilateral and diffuse, rather than lateralized, findings supported by the studies mentioned above. Most marked differences were in limbic and paralimbic regions (bilateral medial temporal lobes, amygdala, hippocampus, parahippocampal gyrus, insula, and cingulate cortices), with abnormalities found 2–4 times more frequently in the ANMDARE group, with ORs ranging from 2.7 to 4, supporting the limbic-predominant pattern previously reported in patients with ANMDARE, but also suggesting a paralimbic predominant pattern. Notably, after Bonferroni corrections, the ANMDARE group also exhibited significantly more MRI abnormalities in the right dorsolateral prefrontal cortex, the bilateral parietal and occipital cortices, and the left lateral temporal cortex, supporting an extralimbic predominant lesional pattern in this population, which, in the present study, we have called the neocortical predominant pattern. Based on our findings, T2-FLAIR abnormalities can be divided into 3 main patterns: limbic (56.5%), paralimbic (55.5%), and neocortical (67%). However, as shown in Table 3, the discriminatory MRI findings between groups were true when comparing the ANMDARE group to the subset of patients with primary psychiatric disorders, but not to the subset of patients with CSF-negative probable autoimmune encephalitis.

More recently, Khabit et al. described a similar group of MRI patterns in a retrospective cohort study. Of 255 patients with ANMDARE, 37 (14.5%) had limbic hyperintensities, and 41 (16.1%) had extralimbic lesions; 10 patients had overlapping demyelinating syndromes. Importantly, this study described, for the first time, the nature of the lesions, incorporating three types of extralimbic lesions: MS-like lesions, extensive lesions, and poorly demarcated fluffy lesions, either multifocal or involving the cerebral cortex or cerebellum [10]. While the nature of MRI abnormalities was not assessed in the present study, our findings regarding abnormalities in limbic, paralimbic, and neocortical regions reinforce both a limbic-predominant pattern and an extralimbic-predominant pattern in ANMDARE as described in previous studies. Importantly, in our sample, patients with overlapping demyelinating syndromes were absent.

It is worth noting that patients with antibody-negative autoimmune encephalitis behave similarly to patients with ANMDARE with respect to MRI abnormalities. As shown in Table 3, when the ANMDARE group and the probable autoimmune encephalitis group were compared, no significant differences were found in most limbic, paralimbic, and neocortical areas. However, there were trends for higher MRI abnormalities in the left parietal, right occipital, and left occipital cortices in patients with ANMDARE, possibly reflecting a slightly more posterior neocortical involvement compared with the probable EA group. Interestingly, previous MRI studies have not documented abnormalities in the posterior cortices (parietal and occipital lobes), but this is consistent with well-established metabolic abnormalities in ANMDARE [16,17,18,19].

Another significant MRI abnormality found in the present study was the high frequency of meningeal enhancement in ANMDARE, approximately 3 times that in controls. Although most reviews on autoimmune encephalitis do not consider meningeal enhancement as an MRI finding seen in this population, and others only consider it to be a “rare” finding [20], the documentation of meningeal enhancement in ANMDARE has been described since the pioneering studies. For example, in the first 100 patients with ANMDARE described by Dalmau et al., 14 patients had enhanced contrast of the overlying meninges [21]. It is crucial to remember that although meningeal enhancement may suggest different etiologies, it has also been observed in ANMDARE and other autoimmune encephalitides and is not as rare as previously suggested. In fact, our sample, meningeal enhancement was observed in both autoimmune groups (26–28%) but was virtually absent in primary psychosis (1.3%). To our knowledge, there is no pathophysiological explanation for the predominant involvement of the pachymeninges compared to the leptomeninges in ANMDARE. Suzuki et al. speculated that the dura mater may be one of the first sites of inflammation in ANMDARE, as it lacks a blood–brain barrier [22]. Further studies are necessary to clarify this hypothesis.

### 4.3. The Clinical Significance of MRI Abnormalities in ANMDARE

Some brain MRI abnormalities have been associated with clinical and prognostic implications; however, most of these findings have not been replicated and remain controversial. Iizuka et al. examined patterns of cerebral and cerebellar atrophy in ANMDARE. The authors found that, in contrast to diffuse cerebral atrophy, cerebellar atrophy was progressive and irreversible and associated with long-term poor clinical outcome [23]. Other studies have found a small but significant volume loss in the total brain volume, cerebellar volume, and brainstem volume [24]. In a study of 382 patients, Balu et al. included brain MRI abnormalities as a criterion for the One-Year Functional Status (NEOS) anti-NMDAR encephalitis score. They found that an abnormal MRI was an independent predictor of poor functional status at 1 year [11]. Conversely, Gabilondo et al. did not find a significant association between the frequency of MRI abnormalities and the likelihood of relapse [25]. Our study does not provide a long-term follow-up to analyze the prognostic implications of MRI abnormalities.

### 4.4. Strengths and Limitations

Blinding expert neuroradiologists to the final diagnosis of patients’ brain MRIs allows a less biased evaluation. Also, a systematic assessment avoids overlooking essential points that are often missed in the casual examination performed in clinical practice. On the other hand, in our study, the control group was heterogeneous, formed by two main diagnostic categories. However, our results are part of a real clinical scenario in which the use of the Graus criteria, the autoimmune psychosis criteria, and the concept of red flags is involved. Importantly, the MRI protocol used in the day-to-day clinical practice at our center to evaluate patients with suspected encephalitis does not include contrast-enhanced FLAIR imaging, which may be more sensitive for evaluating leptomeningeal enhancement. This may have underestimated the presence of this finding in our sample [26]. Due to financial limitations, the study does not provide data about other antineuronal antibodies, such as GAD65-IgG1, GABABR-IgG1, LGI1-IgG4, CASPR2-Ig4, MOG-IgG1, AMPAR-IgG1, DPPX-IgG1, IgG4, mGluR- IgG1, mGluR5-IgG1, D2R-IgG, AK5-Ig, and others [27]. We should consider that the lack of access to specialized services in Mexico could increase the delay between symptom onset and admission to specialized health care, leading to more severe forms of encephalopathy and, therefore, to a more severe clinical expression as compared to other samples.

## 5. Conclusions

In summary, patients with ANMDARE showed a widespread pattern of MRI abnormalities, supporting limbic, paralimbic and neocortical patterns, most strongly affecting the medial temporal structures, the cingulate gyrus, the insula, and the parietal and occipital cortices. In contrast to previous studies, meningeal enhancement was frequent in our ANMDARE group. Although most MRI abnormalities differentiated patients with ANMDARE from the group of primary psychiatric disorders, they did not distinguish them from other antibody-negative autoimmune encephalitis. Based on our findings, T2-FLAIR-MRI abnormalities in patients with ANMDARE can be divided into 3 main, non-exclusive imaging patterns—limbic, paralimbic, and neocortical—and meningeal enhancement should be considered as a feature of autoimmune encephalitis. Further studies are needed to validate these neuroimaging patterns and their clinical implications.

## Figures and Tables

**Table 1:** Clinical and demographic variables in neuropsychiatric patients with and without anti-NMDA receptor encephalitis: focus on psychopathological, neurological and diagnostic data.

Variable	Patients with Anti-NMDAR Encephalitis N = 115	Control Group N = 115	+LR	95% CI	*p*-Value
Neuropsychiatric disturbances					
Hallucinations	76 (66.1%)	60 (52.2%)	1.27	1.02–1.58	0.032
Delusions	76 (66.1%)	75 (65.2%)	1.01	0.84–1.22	0.890
Manic syndrome	27 (23.5%)	22 (19.3%)	1.23	0.74–2.02	0.441
Depressive syndrome	20 (17.4%)	21 (18.4%)	0.95	0.55–1.66	0.839
Suicidal behavior	14 (12.3%)	15 (13.0%)	0.93	0.47–1.84	0.862
Psychomotor agitation	79 (68.7%)	34 (29.6%)	2.32	1.71–3.16	<0.001 *
Severe cognitive dysfunction	93 (80.9%)	59 (51.3%)	1.58	1.29–1.92	<0.001 *
Catatonia	76 (66.1%)	31 (27.0%)	2.45	1.77–3.40	<0.001 *
Neurological disturbances					
Seizures	70 (60.9%)	12 (10.4%)	5.83	3.35–10.16	<0.001 *
Dyskinesia	71 (61.7%)	21 (18.3%)	3.38	2.24–5.11	<0.001 *
Autonomic abnormalities	61 (53.0%)	19 (16.5%)	3.21	2.06–5.01	<0.001 *
Complications					
ICU requirement	34 (29.6%)	9 (7.8%)	3.78	1.90–7.52	<0.001 *
Coma state	27 (23.5%)	5 (4.4%)	5.40	2.16–13.53	<0.001 *
Death	8 (7.0%)	0 (0%)	∞	not estimable	0.004
Diagnostic studies					
Abnormal EEG	99/113 (87.6%)	40/96 (41.7%)	2.10	1.64–2.69	<0.001 *
Abnormal CSF	67 (58.3%)	18 (15.7%)	3.72	2.37–5.85	<0.001 *
Abnormal MRI	65 (56.5%)	31 (27.0%)	2.10	1.49–2.95	<0.001 *

* Significant after Bonferroni correction for multiple comparisons (0.05/17 = 0.0029).

**Table 2:** Analysis of MRI abnormalities in patients with definite anti-NMDA receptor encephalitis versus the control group.

Variable	Anti-NMDAR Encephalitis Group, N = 115	Control Group, N = 115	+LR	95% CI	*p*
Abnormalities in limbic and paralimbic structures					
Medial temporal lobe	64 (55.7%)	30 (26.1%)	2.13	1.51–3.02	<0.001 *
Right amygdala	41 (35.7%)	21 (18.3%)	1.95	1.23–3.09	0.003
Left amygdala	44 (38.3%)	20 (17.4%)	2.20	1.39–3.49	<0.001 *
Right hippocampus	51 (44.3%)	26 (22.6%)	1.96	1.32–2.91	<0.001 *
Left hippocampus	53 (46.1%)	26 (22.6%)	2.04	1.38–3.02	<0.001 *
Right parahippocampal gyrus	27 (23.5%)	12 (10.4%)	2.25	1.20–4.22	0.008
Left parahippocampal gyrus	32 (27.8%)	19 (16.5%)	1.68	1.02–2.79	0.039
Right insular cortex	36 (31.3%)	15 (13.0%)	2.40	1.39–4.14	0.001
Left insular cortex	46 (40.0%)	18 (15.7%)	2.56	1.58–4.13	<0.001 *
Right anterior cingulate cortex	51 (44.3%)	19 (16.5%)	2.68	1.70–4.25	<0.001 *
Left anterior cingulate cortex	49 (42.6%)	20 (17.4%)	2.45	1.56–3.85	<0.001 *
Right middle cingulate cortex	31 (27.0%)	14 (12.2%)	2.21	1.25–3.94	0.005
Left middle cingulate cortex	28 (24.3%)	13 (11.3%)	2.15	1.18–3.94	0.010
Right posterior cingulate cortex	28 (24.3%)	9 (7.8%)	3.11	1.54–6.30	0.001
Left posterior cingulate cortex	26 (22.6%)	10 (8.7%)	2.60	1.31–5.14	0.004
Abnormalities in neocortical structures					
Right dorsolateral prefrontal cortex	44 (38.3%)	20 (17.4%)	2.20	1.39–3.49	<0.001 *
Left dorsolateral prefrontal cortex	42 (36.5%)	21 (18.3%)	2.00	1.27–3.15	0.002
Right orbitofrontal cortex	24 (20.9%)	10 (8.7%)	2.40	1.16–4.97	0.009
Left orbitofrontal cortex	25 (21.7%)	8 (7%)	3.13	1.47–6.64	0.001
Right lateral temporal cortex	39 (33.9)	25 (21.7%)	1.56	1.01–2.40	0.039
Left lateral temporal cortex	49 (42.6%)	21 (18.3%)	2.33	1.50–3.63	<0.001 *
Right parietal cortex	45 (39.1%)	18 (15.7%)	2.50	1.54–4.05	<0.001 *
Left parietal cortex	44 (38.3%)	16 (13.9%)	2.75	1.65–4.58	<0.001 *
Right occipital cortex	46 (40.0%)	11 (9.6%)	4.18	2.28–7.66	<0.001 *
Left occipital cortex	42 (36.5%)	12 (10.4%)	3.50	1.95–6.30	<0.001 *
Abnormalities in other subcortical structures					
Right thalamus	6 (5.0%)	0 (0%)	∞	Not estimable	0.013
Left thalamus	7 (6.1%)	2 (1.7%)	3.50	0.74–16.49	0.089
Right striatum	7 (6.1%)	1 (0.9%)	7.00	0.88–55.99	0.031
Left striatum	8 (7.0%)	1 (0.9%)	8.00	1.02–62.94	0.017
Brainstem	8 (7.0%)	1 (0.9%)	8.00	1.02–62.94	0.017
Cerebellum	10 (8.7%)	1 (0.9%)	10.00	1.30–76.86	0.005
Meningeal abnormalities					
Pachymeningeal enhancement	30 (26.1%)	12 (10.4%)	2.50	1.35–4.64	0.002
Leptomeningeal enhancement	5 (4.3%)	0 (0%)	∞	Not estimable	0.024

* Significant after Bonferroni correction for multiple comparisons (0.05/34 = 0.0014).

**Table 3:** MRI abnormalities in patients with anti-NMDA receptor encephalitis as compared to patients with autoimmune encephalitis NMDAR negative and primary psychiatric disorders.

Variable	Group (A) Anti-NMDAR Encephalitis Group, N = 115	Group (B) Autoimmune Encephalitis Without NMDAR Antibodies, N = 40	Group (C) Primary Psychotic Disorders, N = 75	*p*
Limbic abnormalities				
Medial temporal lobe	65 (56.5%)	19 (47%)	11 (14.7%)	A vs. B, *p* = 0.324 A vs. C, *p* < 0.001
Left amygdala	44 (38.3%)	12 (30%)	8 (10.7%)	A vs. B, *p* = 0.349 A vs. C, *p* < 0.001
Right hippocampus	51 (44.3%)	16 (40.0%)	10 (13.3%)	A vs. B, *p* = 0.633 A vs. C, *p* < 0.001
Left hippocampus	53 (46.1%)	17 (42.5%)	9 (12.0%)	A vs. B, *p* = 0.695 A vs. C, *p* < 0.001
Paralimbic abnormalities				
Left insular cortex	46 (40.0%)	10 (25%)	8 (10.7%)	A vs. B, *p* = 0.089 A vs. C, *p* < 0.001
Right anterior cingulate cortex	51 (44.3%)	11 (27.5%)	8 (10.7%)	A vs. B, *p* = 0.061 A vs. C, *p* < 0.001
Left anterior cingulate cortex	49 (42.6%)	11 (27.5%)	9 (12.0%)	A vs. B, *p* = 0.091 A vs. C, *p* < 0.001
Neocortical abnormalities				
Right dorsolateral prefrontal cortex	44 (38.3%)	9 (22.5%)	11 (14.7%)	A vs. B, *p* = 0.070 A vs. C, *p* < 0.001
Left lateral temporal cortex	49 (42.6%)	12 (30.0%)	9 (12.0%)	A vs. B, *p* = 0.160 A vs. C, *p* < 0.001
Right parietal cortex	45 (39.1%)	11 (27.5%)	7 (9.3%)	A vs. B, *p* = 0.187 A vs. C, *p* < 0.001
Left parietal cortex	44 (38.3%)	8 (20.0%)	8 (10.7%)	A vs. B, *p* = 0.035 A vs. C, *p* < 0.001
Right occipital cortex	46 (40.0%)	7 (17.5%)	4 (5.3%)	A vs. B, *p* = 0.010 A vs. C, *p* < 0.001
Left occipital cortex	42 (36.5%)	8 (20.0%)	4 (5.3%)	A vs. B, *p* = 0.054 A vs. C, *p* < 0.001
Meningeal enhancement				
Pachymeningeal enhancement	30 (26.1%)	11 (27.5%)	1 (1.3%)	A vs. B, *p* = 0.861 A vs. C, *p* <0.001

## Data Availability

The raw data supporting the conclusions of this article will be made available by the authors on request. Requests for access to the data should be directed to: jesus.ramirez@innn.edu.mx.

## Abbreviations

The following abbreviations are used in this manuscript:

ANMDARE	Anti-NMDA receptor encephalitis
CSF	Cerebrospinal Fluid
MRI	Magnetic Resonance Imaging
T2-FLAIR	T2-weighted fluid-attenuated inversion recovery MRI sequence
